# Soil Phosphorus Pools, Bioavailability and Environmental Risk in Response to the Phosphorus Supply in the Red Soil of Southern China

**DOI:** 10.3390/ijerph17207384

**Published:** 2020-10-10

**Authors:** Xiaojun Yan, Wenhao Yang, Xiaohui Chen, Mingkuang Wang, Weiqi Wang, Delian Ye, Liangquan Wu

**Affiliations:** 1 International Magnesium Institute, College of Resources and Environment, Fujian Agriculture and Forestry University, Fuzhou 350002, China; 3170808039@fafu.edu.cn (X.Y.); a199905@163.com (W.Y.); chenxiaohui0914@163.com (X.C.); ye-delian@163.com (D.Y.); 2 College of Resources and Environment, Fujian Agriculture and Forestry University, Fuzhou 350002, China; mkwang@ntu.edu.tw; 3 Institute of Geography, Fujian Normal University, Fuzhou 350007, China; wangweiqi15@163.com

**Keywords:** bioavailability, critical level, phosphorus apparent balance, phosphorus fractions, acidic red soil

## Abstract

Excess phosphorus (P) accumulation in the soil can change the bioavailability of P and increase the leaching risks, but the quantitative evaluation of these responses in acidic red soil is lacking. This study aimed to investigate the composition of soil P fractions under different phosphorus apparent balances (PAB) in acidic red soil and the bioavailability and the leaching change-points of different P fractions. Five phosphorus (P) fertilization rates were applied (0, 16.38, 32.75, 65.50, 131.00 kg P·ha^−1^) in every sweet corn cultivation from the field experiment, and the treatments were marked as P0, P1, P2, P3, and P4, respectively. The PAB showed negative values in P0 and P1 which were −49.0 and −15.0 kg P·ha^–1^ in two years, respectively. In contrast, PAB in P2 as well as in P3 and P4 were positive, the content ranging from 40.2 to 424.3 kg P·ha^−1^ in two years. Per 100 kg ha^−1^ P accumulate in the soil, the total P increased by 44.36 and 10.41 mg kg^−1^ in the surface (0–20 cm) and subsurface (20–40 cm) soil, respectively. The content of inorganic P fractions, including solution phosphate (Sol-P), aluminum phosphate (Al-P), iron phosphate (Fe-P), reduction phosphate (Red-P), and calcium phosphate (Ca-P), significantly increased by 0.25, 16.22, 22.08, 2.04, and 5.08 mg kg^−1^, respectively, in surface soil per 100 kg ha^−1^ P accumulated in the soil. Path analysis showed that the most important soil P fractions contributing to Olsen-P were Sol-P and Al-P, which can directly affect Olsen-P, and their coefficients were 0.24 and 0.73, respectively. Furthermore, the incubation experiments were conducted in the laboratory to investigate the leaching risk of different P fractions, and they showed Sol-P was a potential source of leaching, and the leaching change-points of Al-P and Fe-P were 74.70 and 78.34 mg·kg^–1^, respectively. Continuous P that accumulated in soil changed the composition of P fractions, and the bioavailability as well as the leaching risks increased. This is important in optimizing soil P fertilization management in agricultural ecosystems based on the bioavailability and critical levels for leaching of P fractions.

## 1. Introduction

Phosphorus (P) is one of the most essential nutritional elements for plant growth and development [1]. It plays an important role in maintaining soil fertility and makes the agricultural production system more sustainable. Compared with N and K, P is by far the least mobile and least available to plants in the soil, especially in red soil, which is a nutrient deficient acidic soil that contains low amounts of organic matter and has low water holding capacity [2,3]. In China, the consumption of chemical P fertilizers increased from 1.83 Mt in 1986 to 6.85 Mt in 2015, and the use per area of cropland has also increased steadily since 1986, reaching 50.71 kg P·ha^−1^ in 2015 (Figure S1) [4]. Phosphorus (P) seasonal utilization efficiency is only 11.6% for major cereal crops in China [5], resulting in large amounts of P accumulating in the soil. Excess of P accumulating in the soil has increased the soil P pools [6], and soil P has become an environmental problem, causing the eutrophication of surface water systems [7]. Therefore, it is essential to develop an optimal management strategy for P fertilizer application such as keeping a constant application rate of phosphorus by monitoring Olsen-P in the soil to ensure the maximum P use efficiency in agricultural systems and minimize the environmental risks of P leaching.

The concentration of Olsen-P was employed as a useful indicator for soil P status [8]. However, because Olsen-P in soil is not a single entity, a quantification analysis of the accumulation of soil P into different pools is more useful for predicting the change in soil P status and recommending the P application amount [9]. Two broad categories of soil P pools are inorganic P (Pi) and organic P (Org-P). Inorganic P (Pi) is considered a major source of available P in the soil [10]. In addition, sequential P extraction schemes, such as the methods modified by Chang and Jackson fractionation [11], can be used to further divide Pi into different fractions, including solution phosphate (Sol-P), aluminum phosphate (Al-P), iron phosphate (Fe-P), reduction phosphate (Red-P), and calcium phosphate (Ca-P), according to the differences in major cation bonding to orthophosphate. Red-P consists of Fe-P and Al-P fractions surrounded by an inert coat of another material that prevents the reaction of these phosphates in soil solution and reductant soluble forms which occur with an inert material that may be partially or totally dissolved under anaerobic conditions. With soil P accumulation, a significantly positive linear correlation existed between the change in soil P fractions and the phosphorus apparent balance (PAB), and the PAB was estimated by subtracting the amount of P taken up by plants from the amount of P applied as commercial fertilizer [12]. Related studies in calcareous soil showed that the excessive application of P fertilizer resulted in a substantial accumulation of Ca-P and Al-P [13]. An incubation study indicated that the main Pi fraction in calcareous fertilized soil was Ca-P, while in acidic fertilized soil it was Al-P and Fe-P [14]. Soil types are considered an important factor that affect the response of P fractions to PAB. The composition of P fractions among soil is related to predominant mineral constituents or the size of particles, as well as the degree of soil cultivation [15]. To date, the sweet corn was widely cultivation in acidic red soil; however, there has been no detailed quantitative investigation of the changes in the composition of different P fractions with P accumulation in acidic red soil.

Although the sequential P extraction scheme has made great advances in understanding different soil P fractions, there is a paucity of evidence on the P bioavailability of each fraction. According to the plant bioavailability, P fractions can be regarded as rapidly available P, slowly available P, and unavailable P. Shen et al. [16] used the Pearson correlation coefficients between the Olsen-P and P fractions to suggest their relative bioavailability. However, the correlation of a P fraction with Olsen-P does not necessarily imply a direct contribution to of the soil P fraction on P bioavailability. Some studies have shown that path analysis can provide a better understanding of the processes of P transformation in the soil between different P fractions based on a source–sink relationship to evaluated bioavailability [17]. Long-term culture and fertilization experiments indicated that Al-P and Fe-P have strong effects on Olsen-P in albic soil through path analysis [18]. However, in black soil, the fraction of Fe-P is regarded as a form of slowly available P [19]. The bioavailability of P fractions depends on a range of conditions, such as soil type [20,21]. However, little research has investigated the bioavailability of different P fractions in acidic red soil, which accounts for approximately 30% of the world’s ice-free land [22]. Evaluating these direct and indirect relationships between Olsen-P and P fractions can predict the bioavailability of P fractions by providing quantitative information on the dynamic process of the P replenishing ability.

In addition, previous research studies have reported that the continuous and excessive application of P fertilizer increases the risk of eutrophication by nutrient leaching or entry into rivers and lakes [23]. Several studies have emphasized the risk of excessive Olsen-P values through an analysis of the relationship between Olsen-P and CaCl_2_-P, which can obtain a leaching index to evaluate the critical level for environmental safety of P [24]. However, there is limited information on the critical level for environmental safety of different P fractions. There has been no clear evidence whether other P fractions are at a critical level for leaching through the analysis of the relationship between P fractions and CaCl_2_-P. Thus, this study seeks to remedy these problems by using a method to quantify the leaching change-points for different P fractions.

Understanding the composition of different P fractions in acidic red soil is important in evaluating bioavailability and the critical level for environmental safety, which could be attributed to the optimize soil P fertilization management in agricultural ecosystems. However, little information is available on these topics. Therefore, we explore the soil P fractions under different P treatments in soil through field experiments and incubation experiments. Then we evaluated the link between the Olsen-P and P fractions by path analysis for a better understanding of the bioavailability of P fractions. Meanwhile, leaching change-points of different P fractions and the optimal P supply were determined by soil incubation experiments. This study hypothesized that with the continuation of P application, a considerable effect on the accumulation of P fractions occurred and the bioavailability and critical level for environmental safety changed. The objectives of this study are to quantify the P fraction changes with P accumulation, determine the bioavailability of different P fractions, and determine the leaching change-points for optimal soil P fractions managements.

## 2. Materials and Methods

### 2.1. Study Area

A fertilizer experiment has been ongoing since 2017, involving a sweet corn continuous cropping system. This study was conducted in Zhao’an County (117°13′ E, 36°43′ N), Fujian Province, China. This area has a subtropical monsoon climate with a mean annual precipitation of approximately 1290 mm, and the predominant rainfall occurs from June to August, with a mean annual temperature of 22.4 °C. The monthly dynamics of air temperature and precipitation in the study years are shown in Figure S2. The soil type is acidic red soil, the soil classification of which are Ferralsols or Oxisols, based on FAO or USDA, respectively [25]. The basic chemical properties of soil are listed in Table 1. The proportions of sand, silt, and clay are 50.4%, 33.6%, and 16.0%, respectively.

### 2.2. Field Experiment Designs and Soil Samplings

To investigate the composition of soil P fractions under different phosphorus apparent balances (PAB) in acidic red soil and their bioavailability, a field experiment was conducted from August 2017 to July 2019 under sweet corn continuous cultivation, and the five treatments in the study were replicated three times in a randomized block design. The area of each plot was 75 m^2^. Urea, superphosphate, and potassium sulphate were used as the sources of N, P_2_O_5_, and K_2_O fertilizer, respectively, and applied to sweet corn for each growing season. Five phosphorus (P) fertilization rates were applied (0, 16.38, 32.75, 65.50, 131.00 kg P·ha^−1^) at the same as nitrogen (200 kg N·ha^−1^) and potassium (120 kg K_2_O·ha^−1^) fertilizer levels, and the treatments were marked as P0, P1, P2, P3, and P4, respectively. Soil sampling was conducted after harvesting in July 2019. Six soil cores (each 2.50 cm in diameter and a maximum of 60.00 cm in depth) were randomly selected from each plot in S shape at two depths (surface soil (0–20 cm) and subsurface soil (20–40 cm) depths). About 500 g of soil was taken as one soil sample by quartering method and stored in sealed plastic jars for analysis. Air-dried soil was ground to fine powder to pass through a 2-mm sieve for measuring soil pH, Olsen-P, available K, SOC, TN, CaCl_2_-P, free iron and aluminum oxides, Fe- and Al-oxides, and organically bound Fe- and Al- oxides. Then, it passed through a 1-mm sieve for Sol-P, Al-P, Fe-P, Red-P, Ca-P, and Org-P. Treated soil was subjected to chemical analyses as described in Section 2.3.

### 2.3. Soil and Plant Chemical Analyses

Soil pH was measured in a volume ratio (H_2_O) of 1:5 (w/v) using a pH meter [26]. Olsen-P was extracted using 0.5 mol·L^−1^ NaHCO_3_ at pH 8.5 (2.5 g soil, 50 mL solution, shaken for 30 min) [27]. Available K was extracted with ammonium acetate extraction and subsequent flame photometer analysis [28]. The concentrations of SOC and TN were determined using an elemental analyzer. CaCl_2_-P was extracted using 0.01 mol L^−1^ CaCl_2_ (2.5 g soil, 15 mL solution, shaken for 15 min) [29], Free iron and aluminum oxides (Fed, Ald) were extracted using a dithionitecitrate-bicarbonate (DCB) solution. X-ray noncrystalline Fe- and Al-oxides (Feo, Alo) were extracted using an ammonium-oxalate solution followed by shaking under dark conditions for 4 h. Organically bound Fe- and Al- oxides (Fep, Alp) were extracted using a 0.1 M pyrophosphate solution [30]. Org-P was determined by the combustion method and was calculated by subtracting inorganic P from total P [31], and all of these processes were followed by the molybdenum blue colorimetric method. The soil Pi fraction methods carried out in this study combined the sequential extraction methods described by Chang and Jackson [11], Chen et al. [32], and Prajapati et al. [33] to divide them into five fractions: Sol-P, Al-P, Fe-P, Red-P, and Ca-P. The sequential fractionation method is shown in Table 2. P in plant tissues were extracted with the procedure reported by Arifa et al. [34] and analyzed by inductivity coupled plasma optical emission spectrophotometer.

### 2.4. Incubation Experiment

Incubation experiment soil was sampled from the experimental field at the same farm (original soil without previous additions of P). Soil incubations and soil analyses were conducted in the laboratory to investigate the relationship between the concentration of P fractions and PAB (surface soil, soil bulk density is 1.3 g·cm^−3^), and the relationship between CaCl_2_-P and P fractions. Portions of 60.0 g soil samples were put in 200.0 mL incubation vials and pre-incubated at a soil moisture content of 30% (wt/wt) for 7 days (d) at 25 °C. Then, P was added as KH_2_PO_4_ at rates of 0, 7, 20, 59, 177, 354, 531, and 884 mg P·kg^–1^ to investigate the relationship between the concentration of P fractions and PAB. KH_2_PO_4_ was added at rates of 0, 7, 20, 59, 177, and 354 mg P·kg^−1^ to investigate the relationship between CaCl_2_-P and P fractions, and the P fractions and CaCl_2_-P were analyzed as previously described. Then, the soil moisture content was adjusted to 30%. Samples were incubated at a constant temperature (25 °C) for another 30 d. The experiment was conducted in four replicates. The soil incubation method was described by Bai et al. [24].

### 2.5. Statistical Analysis

Phosphorus apparent balance (PAB) was estimated by subtracting the amount of P taken up by sweet corn from the amount of P applied as commercial fertilizer, P taken up by sweet corn was calculated by multiplying the P concentration by the dry weight.

Statistical and correlation analyses were performed using statistical software version SPSS 21.0 (IBM, Armonk, NY, USA). The data total P and P fractions were submitted to variance analysis, and the means were tested by least significant difference (LSD) at 5% probability. Path analysis was conducted to characterize the direct and indirect relationships among Olsen-P and P fractions using the PROCESS V3.4 plugin in SPSS. The relationship between the CaCl_2_-P and P fractions was described entirely by a linear or a split line model using the REG and NLIN procedures, respectively (Sigma plot, version 11.0, London, UK). 

## 3. Results

### 3.1. Phosphorus Apparent Balance with Total P

**Figure 1:**
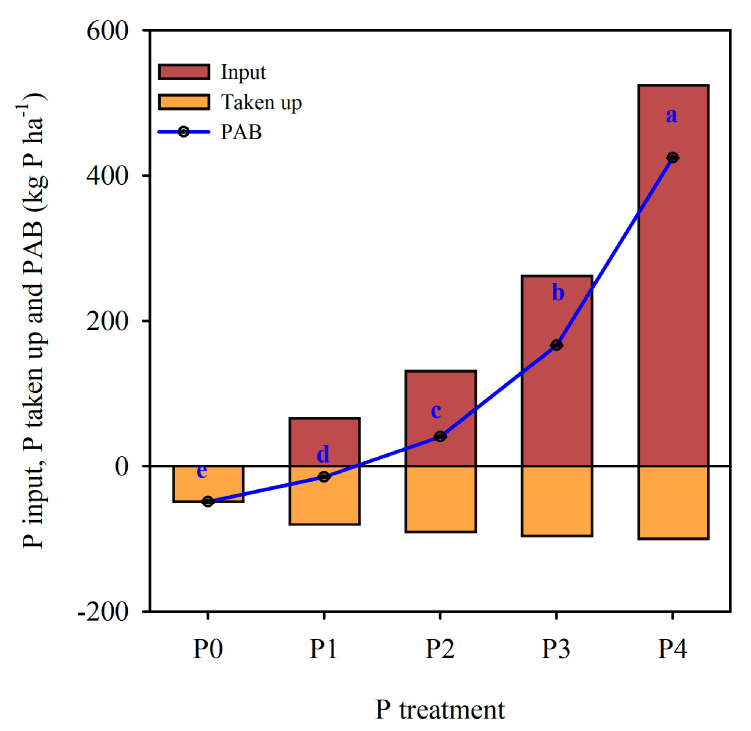
The soil P input, P taken up and phosphorus apparent balances (PAB) from 2017 to 2019 (kg P·ha^−1^). Note: Mean ± Standard deviations, different letters above column indicate significant difference (*p* < 0.05) between various treatment.

Two years of P input, output, and apparent balance (PAB) for different P treatments are listed in Figure 1. The PAB was calculated from the known quantity of P applied and taken up by the harvested crops. The mean PAB was mostly positive in P4 (+424.3 kg P·ha^−1^ in two years) as well as in P3 (+166.1 kg P·ha^−1^ in two years) and P2 (+40.2 kg P·ha^−1^ in two years); these three treatments received excess P beyond the crop requirement. In contrast, the PAB in the P0 and P1 treatments was negative (−49.0 to −15.0 kg P·ha^−1^ in two years); these two treatments P taken up by the crops excess fertilizer applied.

**Figure 2:**
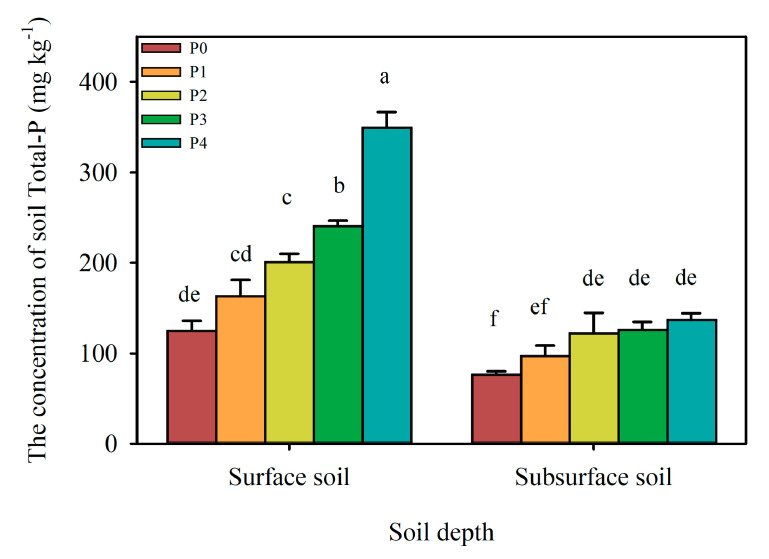
The concentration of soil total P under different P treatments come from field experiment. Note: Mean ± Standard deviations, different letters above column indicate significant difference (*p* < 0.05) between various treatment in different soil depths.

The concentration of total P increased continuously with fertilizer P application (Figure 2). In surface soil (0–20 cm), the concentrations of total P in the P0, P1, P2, P3, and P4 treatments were 124.7, 163.1, 200.2, 239.9, and 348.0 mg·kg^−1^, respectively. In subsurface soil (20–40 cm), the values were 76.3, 97.0, 121.9, 125.6, and 136.6 mg·kg^−1^, respectively. Significant differences were found among different treatments. In surface soil, between P0 and P1 there is lack of a statistically significant difference. The same is for P2 and P1. So, dosages P2-P4 resulted in a significant increase in total P as compared to control (P0), whereas the smallest one (P1) did not. Furthermore, no significant difference was found in subsurface soil among P2, P3, and P4 treatments.

**Figure 3:**
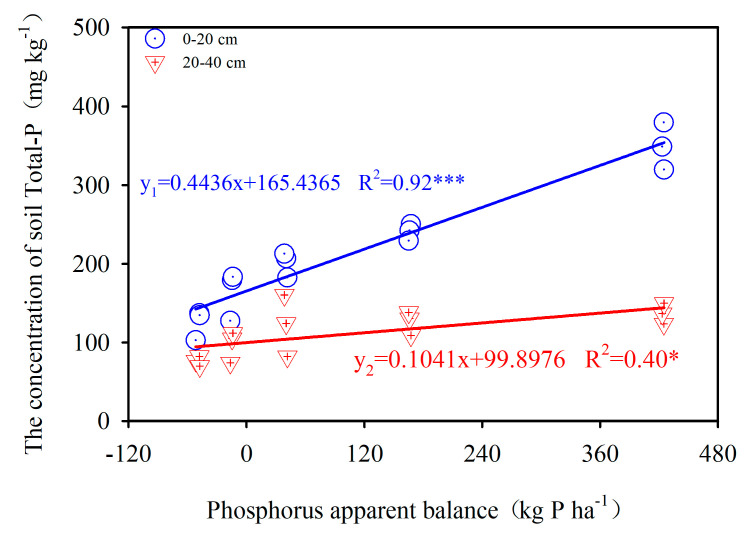
Relationship between PAB and total P come from the field experiment. Note: * means *p* < 0.05, *** means *p* < 0.001.

Significant positive linear relationships between the PAB and the concentration of total P in surface (R^2^ = 0.92, *p* < 0.001) and subsurface (R^2^ = 0.40, *p* < 0.05) soil over the study period (Figure 3) indicate that the change in soil total P can usually be attributed to PAB. For every 100 kg P·ha^−1^ increase in soil PAB, the total P in surface and subsurface soil increased by 44.4 and 10.4 mg·kg^−1^, respectively.

### 3.2. Phosphorus Apparent Balance (PAB) with P Fractions

The concentrations of all Pi fractions increased with increasing of fertilizer P application, but no significant differences were found among Org-P at the same soil depth; however, all P fractions were significantly different under different soil depths (Table 3). In surface soil, compared with the control (P0), the amounts of soil Sol-P, Al-P, Fe-P, Red-P, and Ca-P increased from 60% to 1230%, 153% to 165%, 52% to 361%, 27% to 74%, and 90% to 260%, respectively, with P fertilizer application (P1–P4). The Pi concentrations in subsurface soil was generally much lower than those in surface soil. In subsurface soil, compared with the control, the amounts of soil Sol-P, Al-P, Fe-P, Red-P, and Ca-P with P fertilizer application increased from 114% to 371%, 172% to 348%, 114% to 299%, 42% to 78%, and 14% to 126%, respectively. Furthermore, the recovery rate is about 95% and is shown in Figure S3.

**Figure 4:**
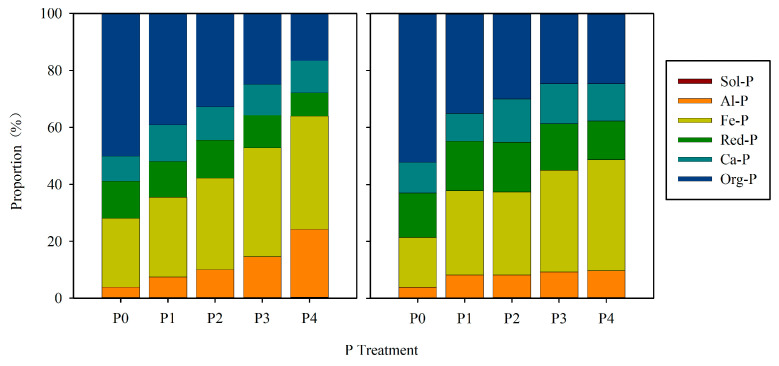
Proportions of various fractions of P in different treatments come from the field experiment (left: surface soil, right: subsurface soil).

The proportion of P fractions with fertilizer P application is shown in Figure 4. In surface soil, where the proportion of Sol-P, Red-P, and Ca-P remained relatively stable after fertilizer P application, the proportion of Org-P declined from 50.1% to 16.6%, while the proportion of Al-P (4–24%), Fe-P (24–40%) relative to the total P increased with increasing fertilizer P application. The subsurface soil showed a similar pattern, and the proportion of Org-P declined from 52% to 25%, while the proportions of Al-P (4–9%) and Fe-P (18–39%) relative to the total P increased with increasing fertilizer P application.

**Figure 5:**
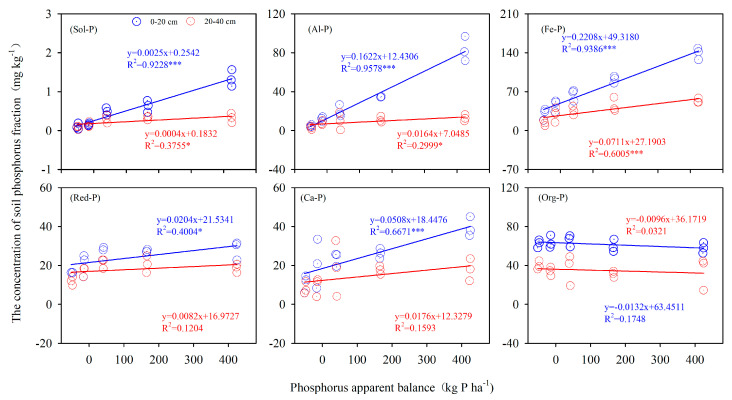
Relationship between PAB and soil P fractions comes from the field experiment. Note: * means *p* < 0.05, *** means *p* < 0.001.

The slopes of the linear regression lines indicate the rate of soil P fraction response to the P apparent balance (PAB) (Figure 5). In surface soil, for each 100 kg P·ha^−1^ surplus, the highest increase in P pool took place in Fe-P fraction (22.08 mg·kg^−1^) followed by Al-P (16.22 mg·kg^−1^), Ca-P (5.08 mg·kg^−1^), Red-P (2.04 mg·kg^−1^), and Sol-P (0.25 mg·kg^−1^). Compared with surface soil, the accumulation rates of different fractions in the subsurface were expressed as Fe-P > Al-P > Sol-P.

**Figure 6:**
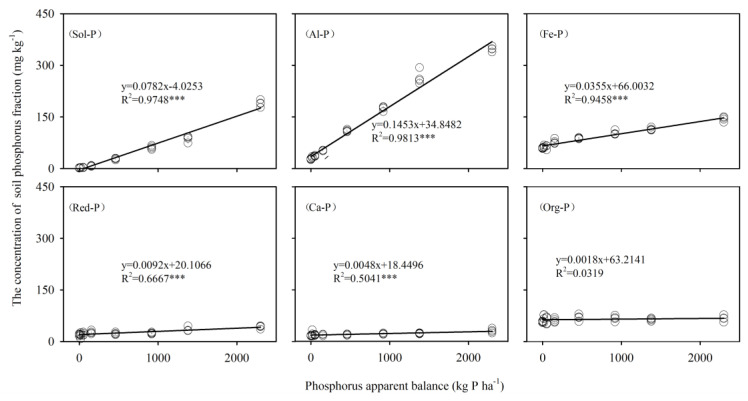
Relationship between PAB and soil P fractions in soil incubation. Note: *** means *p* < 0.001.

Without considering the amount of P from output processes, such as plant uptake, erosion, and leaching losses, through incubation experiment we found that the rate of increase of Al-P and Sol-P was higher than that of Fe-P with soil P accumulation. A positive trend of cumulative PAB in soil incubations caused a linear increase in the concentration of soil Pi fractions (Figure 6). Each 100 kg·ha^−1^ PAB increased in the soil incubations, followed by 14.53 mg·kg^−1^ for Al-P, 7.82 mg·kg^−1^ for Sol-P, 3.55 mg·kg^−1^ for Fe-P, 0.92 mg·kg^−1^ for Red-P, and 0.48 mg·kg^−1^ for Ca-P. When the PAB surpassed 283 kg·ha^−1^, the largest proportion of the P fraction changed from Fe-P to Al-P.

### 3.3. The Bioavailability of P Fractions

**Figure 7:**
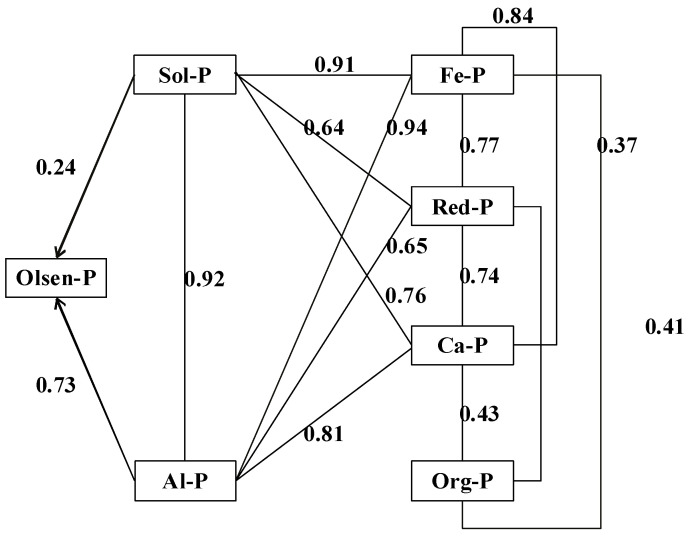
Direct and indirect coefficients between P fractions and Olsen-P.

Square of correlation showed that Olsen-P was positively correlated with all Pi fractions (Table 4). According to square of correlation, fractions can be grouped in a following order: Al-P, Sol-P, Fe-P, Ca-P, and Red-P (R^2^ = 0.98, 0.94, 0.93, 0.79, 0.61, respectively). Moreover, path analysis was used to partition the direct and indirect effects between various soil P fractions and Olsen-P (Figure 7). Direct path coefficients measured the direct effect of each P fraction on Olsen-P, while the indirect path coefficients specified the effect of designated P fractions passed through other P fractions. A high path coefficient indicates a strong influence on Olsen-P, and vice versa. The direct path coefficient showed that the most important soil P fractions for Olsen-P were Sol-P and Al-P, which can directly affect Olsen-P, and their coefficients were 0.24 and 0.73, respectively. Fe-P, Red-P, and Ca-P affect Olsen-P by other fractions first, such as Sol-P and Al-P. The indirect coefficients of Fe-P, Red-P, and Ca-P affecting Olsen-P through Sol-P were 0.91, 0.64, and 0.76, respectively, and those through Al-P were 0.94, 0.65, and 0.81, respectively. 

### 3.4. The Critical Level for Environmental Safety of Phosphorus Fractions

**Figure 8:**
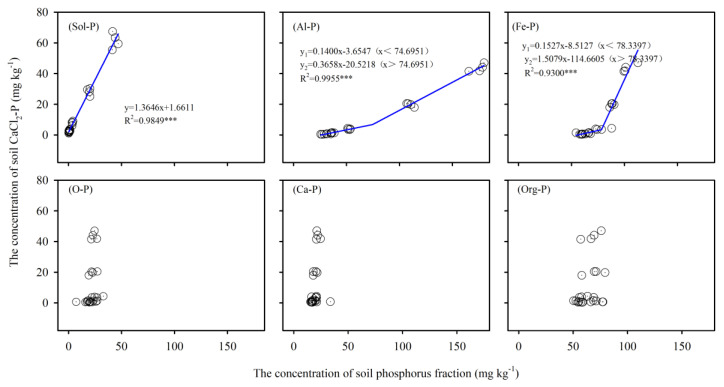
Relationship between soil P fraction and CaCl_2_-P come from incubation experiment. Note: *** means *p* < 0.001.

The critical P level at which the leaching of phosphorus may occur has been defined as the change point and linked to soil P fractions. The relationship between Sol-P and CaCl_2_-P can be described as a linear model. Each 100 mg·kg^−1^ Sol-P increase results in a CaCl_2_-P increase of 136.46 mg·kg^−1^ (Figure 8 Sol-P). The two-segment model described the relationship between soil Al-P, Fe-P, and CaCl_2_-P well (Figure 8 Al-P, Fe-P, R^2^ = 0.99 and 0.93, respectively). Exceeding the inflection point, CaCl_2_-P increased dramatically with increasing soil Al-P and Fe-P. The leaching change-points of soil Al-P and Fe-P were 74.70 and 78.34 mg·kg^−1^, respectively. When the Al-P fractions were below and exceeded the inflection points, for every 100 mg·kg^–1^ increase in soil Al-P, CaCl_2_-P increased by 14.00 and 36.58 mg·kg^−1^, respectively. When the Fe-P contents were below and above the inflection point, for every 100 mg·kg^−1^ increase in soil Fe-P, CaCl_2_-P was 15.27 and 150.79 mg·kg^−1^ higher, respectively. Critical CaCl_2_-P was much higher in Al-P (6.80 mg·kg^−1^) than in Fe-P (3.45 mg·kg^−1^). The ratios of the regression coefficients of the two curves were 2.61 and 9.87 in Al-P and Fe-P, respectively. 

## 4. Discussion

### 4.1. Phosphorus Apparent Balances (PAB), Soil Total P and P Fractions

The P input varied from 0 to 524 kg P·ha^−1^ in the study period; however, the P uptake by sweet corn was approximately 83 kg P·ha^−1^ in the study period. Thus, a large amount of P accumulated in the soil. Quantifying the relationship between total P and P accumulation in the soil can be used to build a model to predict the changes in soil total P with soil PAB [35]. The positive linear correlation of soil total P concentration and soil PAB has been reported in long-term upland field trials, which indicated that most of the P accumulated within the soil profile when the applied P was greater than the required plant P [36]. In this study, for every 100 kg P·ha^−1^ surplus, the total P in surface soil increased by only 44.4 mg kg^−1^, which was lower than that reported in other studies (e.g., 67 mg·kg^−^^1^ in paddy soil) [37]. These differences are influenced by the relatively high sandy content in this study (50.4%). The P adsorption capacity of sand is low because of the smaller surface area [38]. In addition, the pH of the background soil was weakly acidic (pH: 5.90), which has a relatively lower P adsorption capacity [39]. In addition, the P adsorption capacity was positively correlated with the content of soil organic matter [40]. Soil organic matter can provide P sorption sites through complex humic-Al/Fe compounds [41]. In this study, the content of soil organic matter was approximately 8.28 g·kg^−1^, which is lower than that of agricultural land [42]. Kang et al. [43] reported that a unit content increase in soil organic matter can increase the P adsorption capacity by a factor of 0.70 when the content of organic matter is lower than 49.00 g·kg^−1^. It appears that the low background pH and organic matter contributed to the low P adsorption capacity in this study. 

Phosphorus fixed in soil was converted into various soil Pi fractions [44]. It has been well documented that the fractions of P in soil can greatly be dependent on soil types and the rates of P fertilizer. Furthermore, bacteria play an important role in P transformation and can make use of various P fractions [45,46]. In this study, with the application of P fertilizer, concentration of all Pi fractions increased at both soil depths. Without manure fertilizer input and straw return, the concentration and proportion of Org-P decreased with successive planting of sweet corn, the same as the result reported by Adetunji [47]. Fe-P became the predominant fraction as the P fertilizer application rate and PAB increased. A high concentration of Fe-P was generally found in non-calcareous soil with heavy fertilization, which was due to a higher intensity of chemical weathering [48]. In this study, the abundance of Fe-P was also due to the high soil Fe content (Figure S4). In addition, a portion of Al-P and Red-P can convert into Fe-P, causing Fe-P to have consistently higher values than other Pi fractions. Among the P sources, superphosphate will lead to more Fe-P formation during different periods of incubation in the soil [49]. The most abundant P fraction was Fe-P in red soil, which was consistent with the results previously reported by Ao et al. [50]. However, the soil incubations showed that Al-P will become the predominant fraction in soil when the application of P is excessive. This result is explained by the Al-P uptake by plants being higher than that of Fe-P in field experiments [51], thus, without P uptake by plants, Al-P becomes the predominant fraction in soil. This finding is consistent with that of Holford [52], who also found that the majority of soil P fractions were Al-P in acidic soil. The current study found that Fe-P and Al-P will become the dominant P fractions with the lower pH and rapid P accumulation in acidic red soil. 

### 4.2. Bioavailability of P Fractions in Red Soil

A part of the P found in soil is present in forms that are unavailable to plants such as Org-P(Sato). In this study, P fertilizer applied to soil was distributed into different pools, high correlations were found among different P fractions and Olsen-P, except for Org-P (Table 4). Further studies through path analysis showed that the direct effects of soil P fractions on Olsen-P were through Sol-P and Al-P, but Fe-P, Red-P, and Ca-P might transform into Olsen-P through Sol-P or Al-P when the soil P cannot meet the plant’s need, and the indirect coefficients were in the order of Fe-P, Ca-P, and Red-P (Figure 8). In accordance with the present result, previous studies in red soil have demonstrated that Sol-P and Al-P have higher bioavailability than Fe-P or Ca-P which was investigated through correlation and path analysis [50]. Fe-P, Ca-P, and Red-P had indirect effects on Olsen-P, which was also reported by Wang et al. [8] in calcareous soil. Sol-P and Al-P presented the greatest liability as plant absorption took place. On the other hand, Fe-P, Ca-P, and Red-P stocks can return to Sol-P and Al-P and provide the nutrients demanded by plants. Because of the different indirect coefficients and bioavailability, Fe-P was noted as being slowly available, and Ca-P and Red-P were regarded as being unavailable P compared with Fe-P [53]. As the main P fractions in red soil, the different bioavailability values of Al-P and Fe-P are possibly due to the Fe compounds having an enhanced and more effective P adsorption than Al compounds because of more active sites for P precipitation [54]. Al-P was the rapidly available P fraction and increased dramatically; this is the reason why the continued P accumulation in the soil has increased the bioavailability of soil P. 

### 4.3. The Critical Level of Environmental Safety of P Fractions in Red Soil

Inflection points in the relationships between Olsen-P and CaCl_2_-P were used to evaluate the potential risk of P leaching from soil in different soil types. The critical Olsen-P value was much higher in acidic soil than in neutral and calcareous soil [24]. However, information on the relationship between different P fractions and CaCl_2_-P is lacking. In this study, a significant linear correlation was found between CaCl_2_-P and Sol-P and the slope is 1.36 (Figure 8 Sol-P), which means Sol-P is a potential source of leaching. The concentration of Sol-P in soil posed a high leaching risk, which was in line with that previously reported by García-Albacete et al. [55]. We also found that Al-P and Fe-P will be considered critical leaching indexes, and the soil P loss risk of Fe-P was lower than that of Al-P. The main leaching risks for P were associated with bioavailability [56]. Sol-P and Al-P are regarded as rapidly available P accompanied by great leaching risks. However, the leaching risks of slowly available P (Fe-P) are worth considerable attention. The ratio of the regression coefficients of the two curves in Fe-P (factor 9.87) was much larger than that in Al-P (factor 2.61). This finding might be explained by the clay contents in the initial soil in this study being 16.0%, and the P affinity constant was positively and significantly correlated with the clay content [57]. In addition, the CaCl_2_-P seemed to have no response to Red-P, Ca-P, and Org-P, which means that those fractions do not represent leaching risks for P loss. The results from this investigation provide useful data for optimizing the P supply and minimizing the P leaching risk. Combining the changes in Sol-P, Al-P, and Fe-P with PAB in the field experiments, the soil has a leaching risk when the PAB exceeds 131.44 kg P·ha^−1^ for Al-P and Fe-P, and at this time, the Sol-P content was only 0.58 mg·kg^−1^. Therefore, the priority to reduce P leaching risk should be to reduce source-related risks, such as reducing fertilizer application [58], and the PAB should be controlled at less than 131.44 kg P·ha^−1^, which is very important for mitigating the environmental risks of P.

## 5. Conclusions

In general, all Pi fractions increased with PAB. Fe-P and Al-P will become the dominant P fractions with the lower pH and rapid P accumulation in acidic red soil. Furthermore, the path analysis well explained the conversion of the P fractions to Olsen-P and showed the bioavailability potential of Sol-P and Al-P in acidic red soil. The accumulation of P fractions also increased the potential for leaching risks. When PAB was below 131.44 kg P·ha^−1^, the scenario was considered to be environmentally safe. Hence, the P fertilization management strategy based on Olsen-P is not completely reliable, and attention should be paid to the relationship among various P fractions, such as Sol-P, Al-P, and Fe-P, which provide necessary conditions for reducing P leaching risks in the future.

## Figures and Tables

**Table 1:** Basic soil physicochemical properties.

Soil Depth	pH	Total–C (g·kg^−1^)	Total–N (g·kg^−1^)	Avai–K (mg·kg^−1^)	Total–P (mg·kg^−1^)	Olsen-P (mg·kg^−1^)	P Fraction (mg·kg^−1^)					
							Sol-P	Al-P	Fe-P	Red-P	Ca-P	Org-P
Surface soil	5.90 ± 0.07	5.20 ± 0.09	0.60 ± 0.03	40.17 ± 1.20	89.40 ± 4.29	8.19 ± 0.75	0.21 ± 0.07	6.02 ± 0.35	22.58 ± 1.22	10.84 ± 0.67	8.26 ± 0.53	38.49 ± 2.53
Subsurface soil	5.86 ± 0.21	2.86 ± 0.11	0.42 ± 0.02	29.86 ± 1.44	60.18 ± 3.16	7.86 ± 0.98	0.01 ± 0.01	3.28 ± 0.35	11.96 ± 0.44	7.86 ± 0.75	5.45 ± 0.46	29.62 ± 2.16

**Table 2:** Sequential Pi fractions based on the method.

Step	Pi Fraction	Extractant	Shaking Time
1	Sol-P	1 mol·L^−1^ NH_4_Cl	30 min
2 ^a^	Al-P	0.5 mol·L^−1^ NH_4_F (pH:8.2)	60 min
3 ^a^	Fe-P	0.1 mol·L^−1^ NaOH	2 h, 16 h stand, 2 h
4 ^a^	Red-P	0.3 M CD ^b^	25 min
5	Ca-P	0.25 mol·L^−1^ H_2_SO_4_	60 min

Note: ^a^: Saturated sodium chloride solution wash after step; ^b^: CD means 0.3 M sodium citrate (20 mL)-dithionite (1.0 g)-1.0 M sodium hydroxide (5 mL).

**Table 3:** Soil P fractions come from field experiment under sequential extractions (mg·kg^−1^).

Soil Depth	Treatment	Sol-P	Al-P	Fe-P	Red-P	Ca-P	Org-P
Surface soil	P0	0.10 ef	4.75 d	30.20 ef	16.27 cd	10.96 cd	62.48 a
	P1	0.16 def	12.00 cd	45.78 de	20.60 bc	20.86 bcd	63.82 a
	P2	0.49 bc	19.61 c	64.66 c	26.64 ab	23.53 bc	65.75 a
	P3	0.63 b	34.47 b	92.00 b	27.46 a	26.19 b	59.78 a
	P4	1.33 a	83.23 a	139.06 a	28.26 a	39.50 a	57.97 a
Subsurface soil	P0	0.07 f	2.88 d	13.39 f	11.95 d	8.20 d	39.90 b
	P1	0.15 def	7.83 d	28.71 ef	17.00 cd	9.37 d	34.04 b
	P2	0.30 cde	9.64 cd	35.73 e	21.32 bc	18.53 bcd	36.71 b
	P3	0.33 cd	11.27 cd	45.03 de	20.60 bc	17.63 bcd	31.03 b
	P4	0.32 cd	12.90 cd	53.40 cd	18.66 c	17.87 bcd	33.73 b
Source of variation							
Treatment		<0.001	<0.001	<0.001	<0.001	<0.01	ns
Soil depth		<0.001	<0.001	<0.001	<0.001	<0.01	<0.001
T × S		<0.001	<0.001	<0.001	ns	ns	ns

Note: Values followed by different letters indicate significant difference (*p* < 0.05) between various treatment in different soil depths. “ns” in ANOVA results indicates no significant difference.

**Table 4:** Pearson correlation coefficients for soil Olsen-P and phosphorus fractions.

Indicator	Olsen-P	Sol-P	Al-P	Fe-P	Red-P	Ca-P	Org-P
Olsen-P	1						
Sol-P	0.94 **	1					
Al-P	0.98 **	0.92 **	1				
Fe-P	0.93 **	0.91 **	0.94 **	1			
Red-P	0.61 **	0.64 **	0.65 **	0.77 **	1		
Ca-P	0.79 **	0.76 **	0.81 **	0.84 **	0.74 **	1	
Org-P	0.33	0.28	0.33	0.38 *	0.41 *	0.4 3 *	1

Note: * means *p* < 0.05, ** means *p* < 0.01.

## Supplementary Materials

The following are available online at https://www.mdpi.com/1660-4601/17/20/7384/s1, Figure S1: The change in chemical P input and use per area of cropland from 1986 to 2015 in China, Figure S2: Monthly dynamics of air temperature and precipitation in the study years in Zhaoan County, Fujian Province, China, Figure S3: Relationship between the sum of all the P fractions and Total-P (H_2_SO_4_-HClO_4_) by analysis, Figure S4: Relationship between soil extractable Al/Fe and Al-P/Fe-P come from field experiment.
