# Supplementary material for: Soil Phosphorus Pools, Bioavailability and Environmental Risk in Response to the Phosphorus Supply in the Red Soil of Southern China
Source: Int J Environ Res Public Health. 2020 Oct 10;17(20):7384. doi: 10.3390/ijerph17207384 (PMC7599965; doi:10.3390/ijerph17207384)
Supplement: Supplementary file 1 [file ijerph-17-07384-s001.pdf]

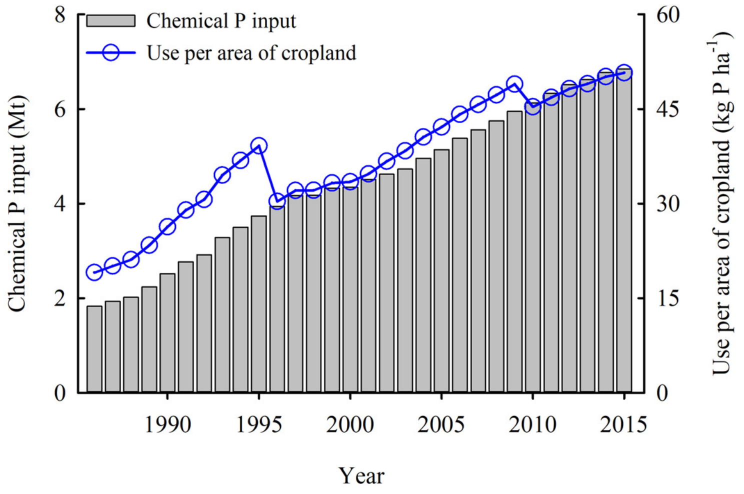

Figure S1. The change in chemical P input and use per area of cropland from 1986 to 2015 in China.

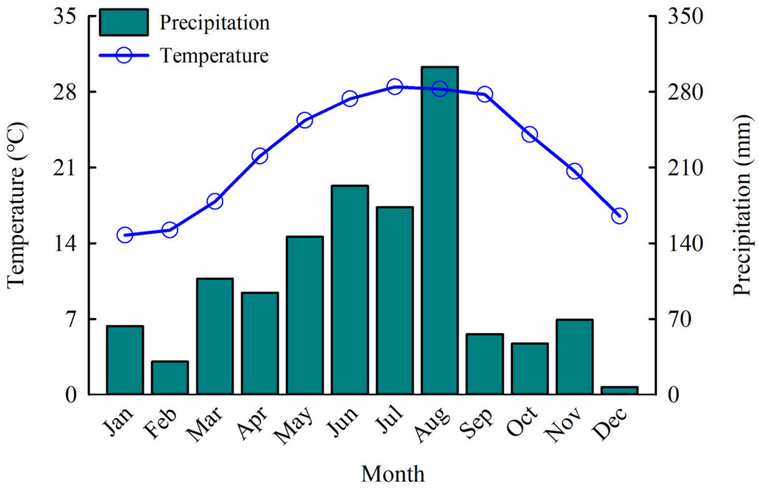

Figure S2. Monthly dynamics of air temperature and precipitation in the study years in Zhaoan County, Fujian Province, China.

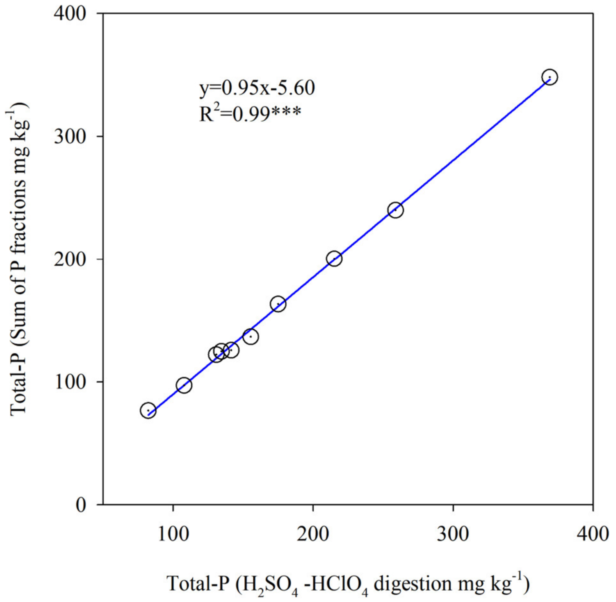

Figure S3. Relationship between the sum of all the P fractions and Total-P (H_2SO_4-$HClO_4$) by analysis.

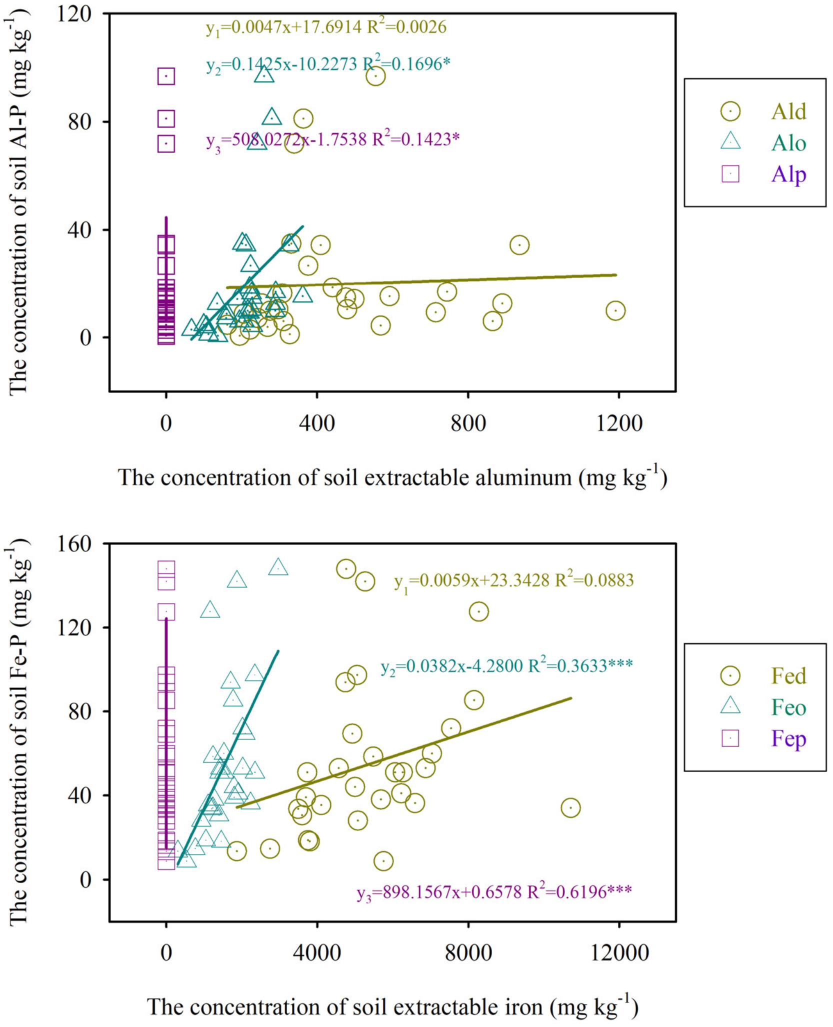

Figure S4. Relationship between soil extractable Al/Fe and Al-P/Fe-P come from field experiment**.** Note: Alp, Fep, Alo, Feo, Ald and Fed refer to organically bound Al oxides, organically bound Fe oxides, non-crystalline Al oxides, non-crystalline Fe oxides, free aluminium and free iron, respectively.
